# Histone deacetylase inhibitors strongly sensitise neuroblastoma cells to TRAIL-induced apoptosis by a caspases-dependent increase of the pro- to anti-apoptotic proteins ratio

**DOI:** 10.1186/1471-2407-6-214

**Published:** 2006-08-24

**Authors:** Annick Mühlethaler-Mottet, Marjorie Flahaut, Katia Balmas Bourloud, Katya Auderset, Roland Meier, Jean-Marc Joseph, Nicole Gross

**Affiliations:** 1 Paediatric Oncology Research, Paediatric Department, University Hospital CHUV, CH-1011 Lausanne, Switzerland; 2 Paediatric Surgery, Paediatric Department, University Hospital CHUV, CH-1011 Lausanne, Switzerland

## Abstract

**Background:**

Neuroblastoma (NB) is the second most common solid childhood tumour, an aggressive disease for which new therapeutic strategies are strongly needed. Tumour necrosis factor-related apoptosis-inducing ligand (TRAIL) selectively induces apoptosis in most tumour cells, but not in normal tissues and therefore represents a valuable candidate in apoptosis-inducing therapies. Caspase-8 is silenced in a subset of highly malignant NB cells, which results in full TRAIL resistance. In addition, despite constitutive caspase-8 expression, or its possible restoration by different strategies, NB cells remain weakly sensitive to TRAIL indicating a need to develop strategies to sensitise NB cells to TRAIL. Histone deacetylase inhibitors (HDACIs) are a new class of anti-cancer agent inducing apoptosis or cell cycle arrest in tumour cells with very low toxicity toward normal cells. Although HDACIs were recently shown to increase death induced by TRAIL in weakly TRAIL-sensitive tumour cells, the precise involved sensitisation mechanisms have not been fully identified.

**Methods:**

NB cell lines were treated with various doses of HDACIs and TRAIL, then cytotoxicity was analysed by MTS/PMS proliferation assays, apoptosis was measured by the Propidium staining method, caspases activity by colorimetric protease assays, and (in)activation of apoptotic proteins by immunoblotting.

**Results:**

Sub-toxic doses of HDACIs strongly sensitised caspase-8 positive NB cell lines to TRAIL induced apoptosis in a caspases dependent manner. Combined treatments increased the activation of caspases and Bid, and the inactivation of the anti-apoptotic proteins XIAP, Bcl-x, RIP, and survivin, thereby increasing the pro- to anti-apoptotic protein ratio. It also enhanced the activation of the mitochondrial pathway. Interestingly, the kinetics of caspases activation and inactivation of anti-apoptotic proteins is accelerated by combined treatment with TRAIL and HDACIs compared to TRAIL alone. In contrast, cell surface expression of TRAIL-receptors or TRAIL is not affected by sub-toxic doses of HDACIs.

**Conclusion:**

HDACIs were shown to activate the mitochondrial pathway and to sensitise NB cells to TRAIL by enhancing the amplitude of the apoptotic cascade and by restoring an apoptosis-prone ratio of pro- to anti-apoptotic proteins. Combining HDACIs and TRAIL could therefore represent a weakly toxic and promising strategy to target TRAIL-resistant tumours such as neuroblastomas.

## Background

Neuroblastoma (NB) is the most frequent solid extracranial tumour in children and is a major cause of death from neoplasia in infancy [1]. These tumours are clinically and biologically heterogeneous, with cell populations differing in their genetic programs, maturation stage and malignant potential [2]. Clinically, spontaneous regressions and tumour maturation are frequent in infants or in low stage tumours, whereas older children often present at diagnosis with high stage progressive and metastatic disease and their overall prognosis is poor [2]. Little improvement in therapeutic options has been made in the last decade, requiring a urgent need for the development of new therapies.

Anti-cancer therapies mediate their cytotoxic effect by predominantly inducing apoptosis in tumour cells. Apoptosis may be induced by triggering the death receptors (extrinsic pathway) or the mitochondria (intrinsic pathway) leading to the activation of effector caspases [3]. Tumour necrosis factor-related apoptosis-inducing ligand (TRAIL) is a promising candidate for therapy of many forms of cancer as it selectively induces cell death in transformed cells, sparing normal tissues [4]. TRAIL mediates apoptosis by activation of the death receptor pathway. Its interaction with TRAIL-R1 and -R2 receptors leads to recruitment of adaptor FADD and initiator caspase-8 to the DISC, resulting in caspase-8 activation and initiation of a cell death cascade by direct cleavage of effector caspases [4,5]. The process is positively regulated and amplified by caspase-3-mediated activation of caspase-8 [6,7], and/or by parallel activation of the mitochondrial pathway via caspase-8-dependent cleavage of Bid [8], resulting in activation of the apoptosome through Bax and Bak oligomerisation and the release of cytochrome-c and Smac/DIABLO into the cytosol. Conversely, negative regulation is promoted by the caspase-8 antagonist c-FLIP [9] or by anti-apoptotic Bcl-2 and Bcl-x_L-_mediated blockade of mitochondria activation [10]. In addition, other inhibitors of apoptosis proteins (IAPs), such as cIAP-1/-2 and XIAP [11] interact with effector caspases, which are neutralized by Smac/DIABLO [3]. Survivin, an other IAP shown to be over-expressed in most tumours, protects cancer cells from apoptosis by interacting with Smac/DIABLO.

Resistance to TRAIL-induced apoptosis in various tumours was described to be caused by the deregulation of diverse signalling molecules such as down-regulation of TRAIL-receptors, caspase-8, caspase-10 or Bax, or over-expression of c-FLIP, Bcl-2, Bcl-x_L _or survivin [12]. In N-type NB cells, resistance to TRAIL was attributed to the down-regulation of caspase-8 expression by hypermethylation or allelic deletion [13-15], as well as to the down-regulation of cell surface TRAIL-R1/-R2 expression [16]. Numerous TRAIL resistant tumour cell lines were reported to be sensitised to TRAIL by combined treatments with chemotherapeutic agents, cycloheximide (CHX), IFN-γ or irradiation by diverse cell-type specific mechanisms [17,18]. We have previously shown that NB cells could be sensitised to TRAIL by subtoxic doses of chemotherapeutic drugs or CHX by the activation of extrinsic and intrinsic apoptotic pathways and caspases-dependent cleavage of XIAP, Bcl-x_L _and RIP [19]. However as chemotherapeutic drugs are non-specifically and highly toxic toward non-tumoral cells, it may be beneficial to develop alternative and less toxic therapeutic strategies that synergise with TRAIL.

Histone deacetylase inhibitors (HDACIs) are a new class of promising anti-cancer agents which inhibit tumour growth both in vitro and in vivo with very low toxicity toward normal cells [20]. Recently, several HDAC inhibitors have entered Phase I and Phase II clinical trials and demonstrate encouraging anti-tumour activity in a variety of cancer types [21]. The anti-tumour effect of HDACIs was proposed to result from accumulation of acetylated histones leading to activation of genes involved in inhibition of tumour cell growth [20]. Altered activities of histone deacetylases or histone acetyl transferases are indeed involved in different human cancer [22,23]. HDACIs mechanism of action appears to involve cell cycle arrest, induction of apoptosis and differentiation both in vitro and in vivo [21,22]. The mechanisms of induction of apoptosis by HDACIs are cell type specific and involve the activation of the intrinsic apoptotic pathways. HDACIs can also synergise with TRAIL to induce apoptosis by various mechanisms depending on tumour types, such as increased expression of TRAIL-receptors and TRAIL [24-28], decrease in c-FLIP [24,26,29], Bcl-x_L _[30], XIAP and Bcl-2 expression (or activation) [31], or increased formation of the DISC [26,32].

This is the first report describing the detailed mechanisms of TRAIL sensitization by HDACIs in neuroblastoma cells. The analysis of the mechanisms by which the three HDACIs, sodium butyrate (NaB), Trichostatin A (TSA) and suberoylanilide hydroxamic acid (SAHA) enhance the action of TRAIL, demonstrates that HDACIs enhance caspases activation and restores a positive ratio between pro- and anti-apoptotic proteins in favour of apoptosis. In addition, we demonstrate that HDACIs also potentiate TRAIL action by increasing the amplitude and the kinetics of the apoptotic process. The association of TRAIL and HDACIs, two non-toxic anti-cancer agents, could be of therapeutic benefit for the treatment of children with NB.

## Methods

### Cell culture and reagents

The caspases-8 positive neuroblastoma cells SH-EP, SH-EP FADD-DN [33], SH-EP-FLIP [34] and SK-N-AS were grown in DMEM medium supplemented with 10 % of FCS, 200 μg/ml gentamicin (Essex Chemie AG). Cells were incubated with indicated amount of soluble recombinant TRAIL (a gift from J. Tschopp) and cross-linking mouse anti-Flag Ab M2 (Sigma) with a constant ratio of 1/5 of TRAIL to M2 respectively. Sodium butyrate (Fluka) was dissolved in H_2_O and stored at -20°C. SAHA (Biovision) and TSA (Sigma) were dissolved in DMSO and store at -20°C. Cells were pretreated 30 min with caspases inhibitors zVAD-fmk (100 μM, Bachem), zVDVAD-fmk (50 μM, Calbiochem), zIETD-fmk, zDEVD-fmk, and zLEHD-fmk (50 μM, R&D systems) before TRAIL or HDACIs treatments.

### Cell viability assays

Cells (1–2.5*10^5^/well in 96-well-plates; 100 μl) were plated 24 h before treatment and incubated with TRAIL and/or HDACIs for 48 h. Assays were performed in quadruplicates. Viability was measured using the MTS/PMS cell proliferation kit from Promega according to manufacturer's instructions. Percentage of cell viability as compared to untreated controls was calculated.

### Measurement of apoptosis by detection of sub-diploid population

Cells were harvested by trypsinization, washed twice with ice-cold PBS, resuspended in 1 ml of ice-cold PBS, and fixed with 3 ml of 100% ice-cold ethanol for 1 h at 4°C. For staining with propidium iodide (PI), cells were washed twice in ice-cold PBS and incubated for at least 30 min at room temperature in 0.2 ml of PBS containing 200 μg/ml RNase A and 10 μg/ml propidium iodide. The stained cells were analyzed using a FACScan flow cytometer (Becton Dickinson).

### Immunoblotting

Whole cell extracts were prepared as already described [14]. Protein extracts (30–50 μg) were loaded on 12% SDS-PAGE and transferred on nitrocellulose membranes. Blots were saturated with 5% skim milk, 0.1 % Tween 20 in TBS and revealed using mouse monoclonal antibodies to detect caspase-8 (MBL), caspase-2 (Apotech corporation), caspase-3, caspase-7, XIAP, RIP, cytochrome-c (BD Pharmingen), AIF (Santa Cruz), β-actin (Sigma). Polyclonal rabbit antibodies were used to detect caspase-9 (Cell Signaling), Bid, Bcl-x_L _(BD Transduction Laboratories), Bim (Imgenex), survivin (R&D systems). Binding of the first antibody was revealed by incubation with either goat anti-mouse IgG (Jackson ImmunoResearch) or goat anti-rabbit IgG (Nordic Immunological Laboratories). Bound antibodies were detected using the Lumi-light western blotting substrate (Roche) according manufacturer's instructions.

### Caspases activities

Caspases-8, -2, -3 and -9 protease activities were measured using the caspases-3, -8, -9 colorimetric protease assay kits from MBL and caspases-2 colorimetric substrate VDVAD-pNA from Alexis. Cytosolic lysates were prepared after TRAIL and/or HDACIs treatments according to manufacturer instructions. One hundred μg of protein extracts were incubated with 200 μM of IETD-pNA, VDVAD-pNA, DEVD-pNA, and LEHD-pNA colorimetric substrates for 3 h at 37°C. Cell lysates were incubated with 10 μM of caspase inhibitor (zIETD-fmk, zVDVAD-fmk, zDEVD-fmk, or zLEHD-fmk) for 30 min before addition of the respective caspase substrate for control experiments. Hydrolysed pNA was detected using a microtiter plate reader at 405 nm. Background absorbance from cell lysates and buffers were subtracted from the absorbance of stimulated and unstimulated samples before calculation of relative caspases activities.

### Analysis of mitochondrial transmembrane potential

The drop of mitochondrial membrane potential ΔΨm was measured by staining the cells with the fluorescent dye JC-1 [35] according to manufacturer's protocol (Calbiochem). Loss of ΔΨm resulting in reduction of red aggregates was measured by flow cytometry using the FL2 channel (550–650 nm) (FACScan, Becton Dickinson). Results are given in percentage of cells with low ΔΨm compared to untreated controls.

### Cell surface immunostaining

Cells were washed in FACS buffer (RPMI, 10 % FCS, 2 mM EDTA) and stained with mouse monoclonal antibodies anti-TRAIL-R1, -TRAIl-R2 and -TRAIL (Alexis), followed by goat secondary antibody conjugated with FITC (Caltag laboratory) and analysed by FACScan (Beckton Dinkinson).

### Transfection with siRNAs

Survivin siRNA targets nucleotides 235–253 of survivin mRNA 5'-AAGGAGCTGGAAGGCTGGGAGTT-3' and control siRNA is composed of the inverse sequence 5'-AAGAGGGTCGGAAGGTCGAGG-3', as described previously [36]. siRNAs were a gift from the lab of Uwe Zangemeister-Wittke. 180'000 cells/well were plated in 12 wells (1 ml) and transfected 8 h later with 100 nM or 25 nM of siRNAs with 3 μl or 2 μl respectively of Lipofectamine2000 according to manufacturer's instructions (Invitrogen). Sixteen h after transfection, cells were plated in 96 wells (10'000 cells/well) and induced 24 h later with TRAIL and/or HDACIs for 48 h.

## Results

### HDACIs strongly sensitise caspases-8 positive NB cell lines to TRAIL-induced apoptosis

**Figure 1:**
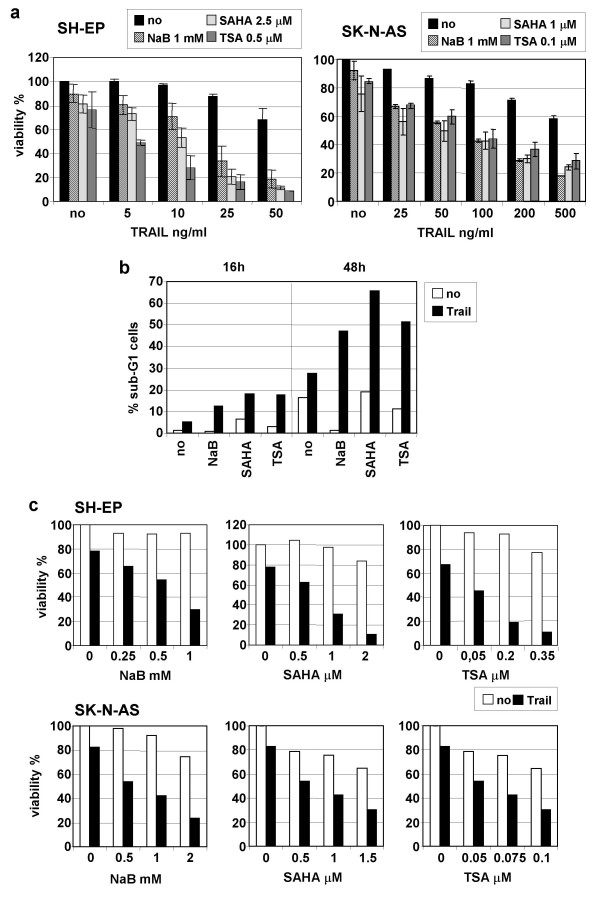
**Subtoxic doses of NaB, SAHA and TSA sensitise NB cells to TRAIL-induced cell death**. **A** Sensitisation is dependent on the concentration of TRAIL. SH-EP and SK-N-AS cells were treated with the indicated amount of TRAIL and NaB, SAHA or TSA for 48 h. Cell death was measured by MTS/PMS cell proliferation assays. Mean values of three or two independent experiments are shown for SH-EP or SK-N-AS cells, respectively. **B** Subtoxic doses of NaB, SAHA and TSA sensitise NB cells to TRAIL-induced cell death. Sub-G1 apoptotic cells were detected by the propidium staining method after stimulation for 16 h or 48 h with 1 mM of NaB, 2.5 μM of SAHA or 0.5 μM of TSA in absence or in presence of TRAIL (25 ng/ml) and M2 (125 ng/ml). **C **Subtoxic doses of NaB, SAHA and TSA sensitise NB cells to TRAIL-induced cell death. Sensitisation is dependent on the concentration of HDACIs. SH-EP cells were treated with TRAIL 50 ng/ml and M2 (250 ng/ml) and SK-N-AS cells with TRAIL 100 ng/ml and M2 (500 ng/ml), and indicated amount of NaB, SAHA or TSA for 48 h.

We have previously shown that caspases-8 positive NB cells are weakly sensitive to TRAIL and could be sensitised by subtoxic concentrations of chemotherapeutic drugs such as doxorubicin or cisplatin [19]. Here we analysed the ability of three different HDACIs belonging to two structural classes, the short-chain fatty acid NaB and the hydroxamic acids SAHA and TSA to sensitise these NB cells to TRAIL-mediated apoptosis. Treatments with subtoxic concentrations of NaB, SAHA and TSA highly increased TRAIL-induced apoptosis in SH-EP cell line as analysed by proliferation assays (Fig. 1a) or by the propidium iodide staining method (Fig. 1b). SK-N-AS cells, which are more resistant to TRAIL-induced apoptosis, could also be strongly sensitised to TRAIL by HDACIs (Fig. 1a). Potentiation of cell death by co-treatment with TRAIL and HDACIs was dependent on both the concentration of TRAIL and HDACIs (Fig. 1a and 1c).

In contrast, HDACIs could not sensitise NB cells to low doses of classical chemotherapeutic drugs, such as Doxorubicin, Taxol, Cisplatin or Etoposide, except for a very weak sensitisation observed when DOX was combined with TSA (data not shown). In addition, HDACIs could not overcome the resistance of caspases-8 positive NB cells to Fas ligand (data not shown), which could in contrast be sensitised by shRNAs-mediated downregulation of c-FLIP_L _[34].

### Sensitisation to TRAIL by HDACIs requires the integrity of the receptor pathway, without involving TRAIL-receptors expression

**Figure 2:**
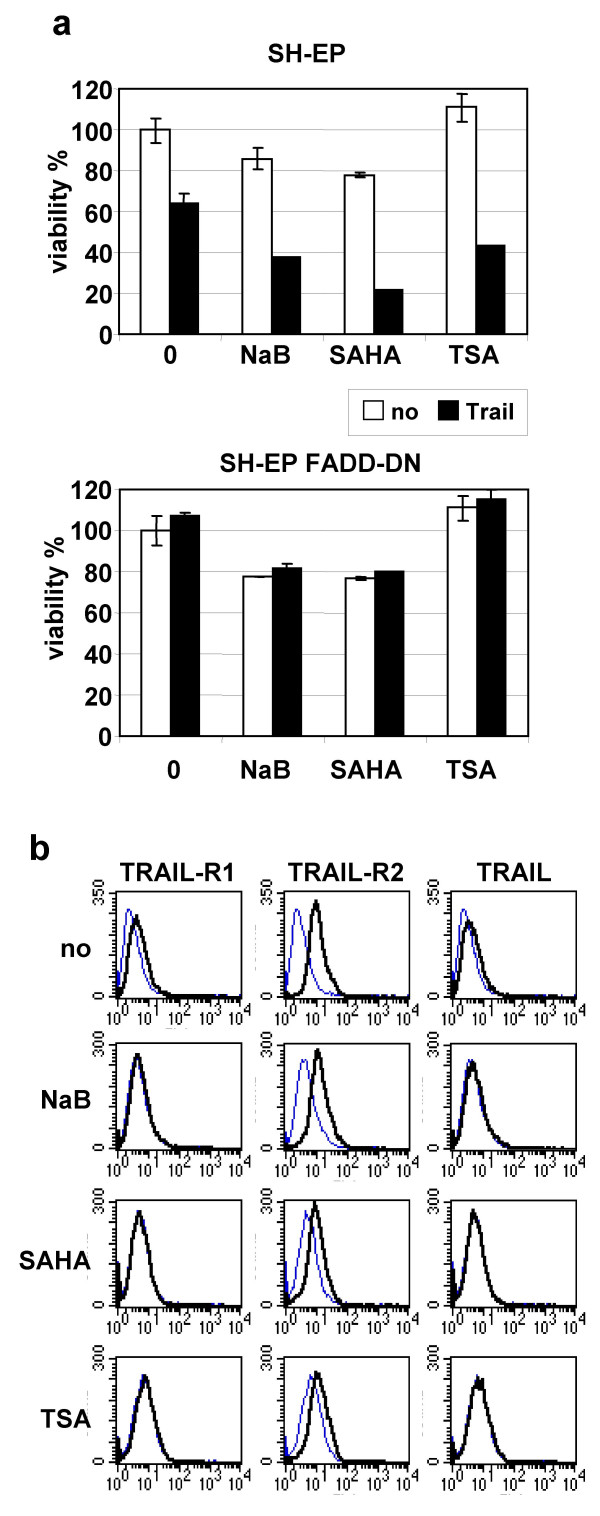
**Sensitisation by HDACIs to TRAIL requires an intact TRAIL receptor pathway**. **A** SH-EP and SH-EP-FADD-DN cells were treated 48 h without TRAIL or with TRAIL (50 ng/ml) and M2 (250 ng/ml) in the presence or absence of NaB (1 mM), SAHA (2.5 μM) or TSA (0.5 μM).**B **No induction in cell surface expression of TRAIL and TRAIL receptors induced by HDACIs in NB cells. SH-EP cells treated for 24 h with NaB (1 mM), SAHA (2.5 μM) or TSA (0.5 μM) were incubated with antibody against TRAIL-R1, TRAIL-R2 and TRAIL (thick line) or without primary antibody (thin line) and then with a FITC labelled secondary antibody.

The contribution of the death receptor pathway in this sensitisation mechanism was analysed using SH-EP cells over-expressing a FADD dominant-negative protein (FADD-DN), lacking the death effector domain required for caspase-8 recruitment to the DISC [33]. SH-EP-FADD-DN were completely resistant to TRAIL and could not be re-sensitised to TRAIL by co-treatment with subtoxic doses of NaB, SAHA or TSA (Fig. 2a). In addition, SH-EP cells over-expressing c-FLIP_L_, and caspase-8/-10 negative N-type NB cells could not be sensitised to TRAIL by combined treatments with TRAIL and HDACIs (data not shown). These findings indicate that the integrity of the TRAIL-receptor pathway is necessary for TRAIL sensitisation by NaB, SAHA and TSA.

To analyse the detailed mechanisms by which these HDACIs enhance the action of TRAIL, the cell surface expression of TRAIL and the TRAIL-receptors was measured by FACS. Indeed, recent studies have described that HDACIs induced the expression of TRAIL and TRAIL-receptors in leukaemia cells, breast and colon cancer cells [24-28], while other reports have shown no changes in the expression level of DR4, DR5 and DcR2 in melanoma and lymphoma cells [31,37]. No increase in the cell surface expression of TRAIL, TRAIL-R1 and TRAIL-R2 and the decoy receptor TRAIL-R3 was observed in SH-EP and CA2E NB cells after 24 h of treatment with sub-toxic doses of NaB, SAHA or TSA (Fig. 2b and data not shown). These results indicate that the sensitising effect of HDACIs on NB cells acts downstream of the death receptors.

### Activation of apoptotic proteins

**Figure 3:**
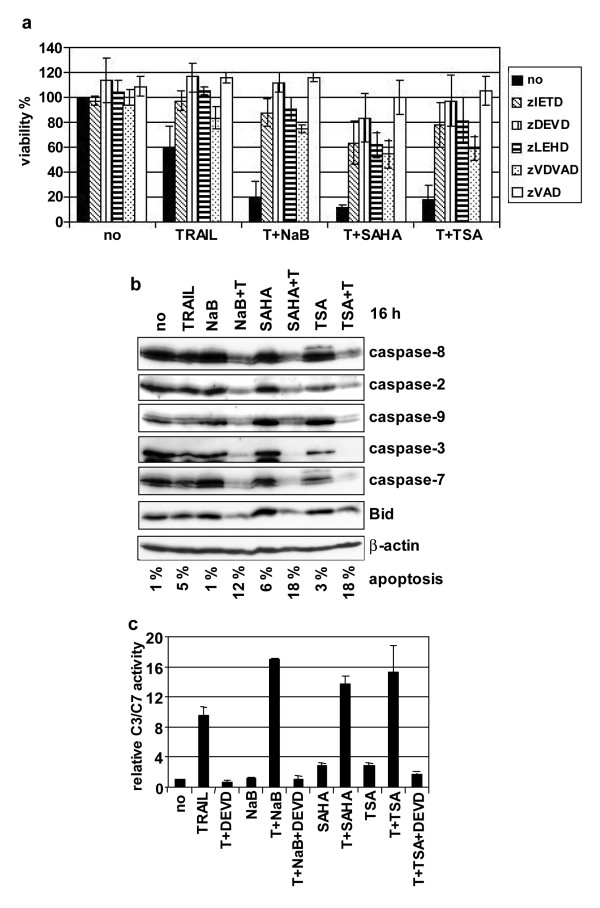
**HDACIs and TRAIL induce caspases-dependent apoptosis**. **A** SH-EP cells were uninduced (no) or induced for 48 h by TRAIL (25 ng/ml) and M2 (125 ng/ml) (T) and NaB (1 mM), SAHA (2.5 μM) or TSA (0.5 μM), without caspases inhibitors, or with zIETD-fmk, zDEVD-fmk, zLEHD-fmk, zVDVAD-fmk or zVAD-fmk. Mean values of three representative experiments are shown. **B** Caspases cleavage is increased by simultaneous treatments with TRAIL and HDACIs. SH-EP cells were untreated (no) or treated with TRAIL (25 ng/ml) and M2 (125 ng/ml) (T) and/or NaB (1 mM), SAHA (2.5 μM) or TSA (0.5 μM) for 16 h. Whole cell extracts were analysed by immunoblotting for the cleavage of caspases-8, -2, -9, -3 and -7, and Bid. β-actin was used as loading control. Percentage of apoptotic cells (sub-G1 population) as analysed by the PI staining method after 16 h of treatments are indicated. **C **Caspase-3-like activities were induced by TRAIL/HDACIs co-treatments. Hydrolysis of DEVD-pNA was measured in SH-EP cells unstimulated (no) or treated with TRAIL (T) and/or NaB, SAHA or TSA for 16 h as in Fig. 3b. The caspase-3-like activities of stimulated cells, relative to unstimulated cells are indicated. The caspase-3/-7 inhibitor DEVD-fmk was used as control to inhibit hydrolysis of DEVD-pNA.

To understand the mechanisms of sensitisation to TRAIL-mediated apoptosis by HDAC inhibitors, the involvement of caspases was analysed using caspases inhibitors. SH-EP cells were strongly protected from apoptosis induced by combined treatment with TRAIL and HDACIs by the caspase-8 (zIETD-fmk), the caspase-3/-7 (zDEVD-fmk), the caspase-9 (zLEHD-fmk), the caspase-2 (zVDVAD-fmk) protease inhibitors and the pan-caspases inhibitor zVAD-fmk (Fig. 3a). This demonstrates that HDACIs-mediated sensitisation to TRAIL is caspases-dependent.

The extent of caspases activation induced by simultaneous addition of TRAIL and HDACIs was further analysed by immunoblotting in SH-EP cells. Co-incubation with TRAIL and NaB, SAHA or TSA for 16 h increased the cleavage of procaspases-8, -2, -3, -7 and -9, compared to treatment with TRAIL alone (Fig. 3b). The observed decrease of Bid was also enhanced by combined treatments (Fig. 3b). This resulted in Bid activation by caspases dependent cleavage as further depicted in figure 5d. In contrast, subtoxic doses of HDACIs alone failed to induce caspases activation or changes in the steady-state level of procaspases-8, -2, -3, -7 and -9, Bid or FADD (Fig. 3b and data not show).

To confirm the increased activation of caspases by combined treatment, the caspase-3-like activity was measured in SH-EP cells. The relative caspase-3-like activities in lysates of cells treated with TRAIL and NaB, SAHA or TSA were superior to that of cells treated with TRAIL alone, whereas no hydrolysis of DEVD-pNA was measured in lysates from cells treated with HDACIs alone (Fig. 3c). These results confirm that the decrease in caspases-3 and -7 as observed in Fig. 3b, results from caspases activation rather than on deregulation of the pro-caspase expression level.

### HDACIs increase the magnitude of caspases activation

**Figure 4:**
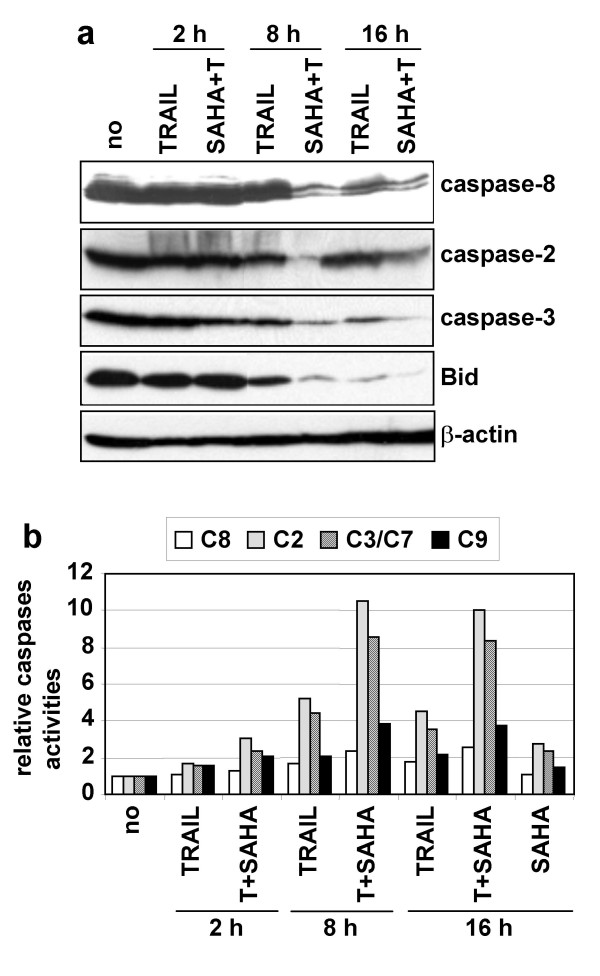
**Time-course analysis of caspases activation**. **A** SH-EP cells were unstimulated (no) or stimulated with TRAIL (25 ng/ml) and M2 (125 ng/ml) (T) alone or in combination with SAHA (2.5 μM) for 2, 8 or 16 h. Whole cell extracts were analysed by immunoblotting for the cleavage of caspases-8, -2, -3, and Bid. β-actin was used as loading control. **B** Time-course analysis of caspases activation. Caspases-8, -2, -3/7 and -9 activities were measured in lysates of SH-EP cells treated as in a) by measuring the hydrolysis of their respective caspases colorimetric substrates. Caspases activities of stimulated cells, relative to unstimulated cells are indicated.

The kinetic of caspases cleavage was then analysed by a time-course experiment. Results show that combined treatments with TRAIL and SAHA increased the cleavage of Bid and procaspases-8, -2 and -3, as cleavages became apparent after 8 h of treatment with TRAIL and SAHA, while only weak cleavages were observed at 8 h with TRAIL alone (Fig. 4a). Similar results were obtained with TRAIL and NaB, although a faster kinetic of activation was observed (data not shown). Then, the kinetic and the extent of caspases-8, -3/7, -2 and -9 activation was measured using colorimetric caspases substrates. Interestingly, the maximal level of caspases activation was reached after 8 h treatment, as no further increase was observed after 16 h (Fig. 4b). In addition, the extent of caspases activities induced by combined treatments was higher than that induced by TRAIL alone. These analyses suggest that HDACIs sensitise NB cells to TRAIL-induced apoptosis by increasing the extent of caspases activation and thereby enhancing the extent of the apoptotic process.

### HDACIs increase the activation of the mitochondrial pathway induced by TRAIL

**Figure 5:**
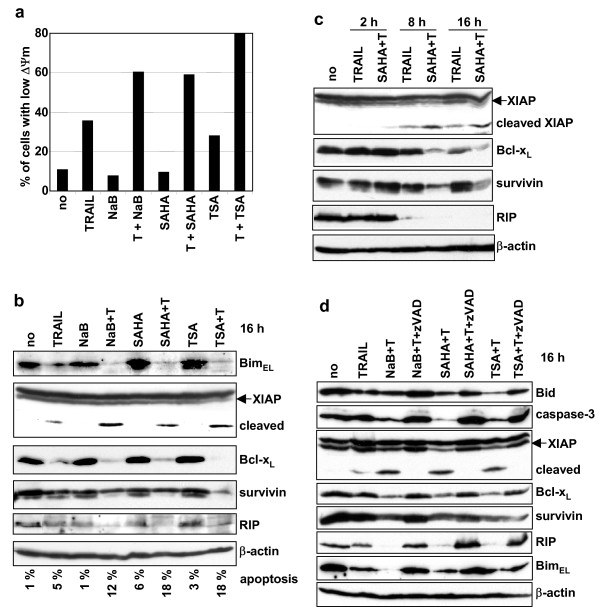
**Activation of the mitochondrial signalling pathway**. **A** SH-EP cells were untreated (no) or treated with TRAIL (50 ng/ml) and M2 (250 ng/ml) (T) and/or NaB (1 mM), SAHA (2.5 μM) or TSA (0.5 μM) for 20 h. The loss of ΔΨm was measured using the fluorescent probe JC-1. The percentage of cells with low ΔΨm is indicated. **B **Simultaneous treatments with TRAIL and HDACIs induce inactivation of anti-apoptotic proteins. SH-EP cells were treated as described in Fig. 3b. Percentage of apoptotic cells (sub-G1 population) as analysed by the PI staining method after 16 h of treatments are indicated. **C **Time-course analysis of anti-apoptotic protein cleavage. SH-EP cells were treated as in Fig. 4a. **D **Co-treatments with TRAIL and HDACIs induce caspases-dependent cleavages of pro- and anti-apoptotic proteins. SH-EP cells were untreated or treated with TRAIL (25 ng/ml) and M2 (125 ng/ml), or with TRAIL (T)+NaB (1 mM), T+SAHA (2.5μM) or TSA (0.5 μM) in the presence or absence of zVAD for 16 h. b, c, and d. Whole cell extracts were analysed by immunoblotting for the presence of different pro- and anti-apoptotic proteins. β-actin was used as loading control.

The observation of an increased cleavage of Bid and caspase-9, as well as the protection by the caspase-9 inhibitor from cell death induced by co-treatment with TRAIL and HDACIs, suggested the involvement of the mitochondrial apoptotic pathway. To analyse this hypothesis, we measured the disruption of the transmembrane mitochondrial potential (ΔΨm) using the fluorescent dye JC-1 in SH-EP cells after TRAIL/HDACIs treatments. TRAIL alone induced a drop of the ΔΨm in 35% of the cells after 20 h of induction, whereas co-treatments with NaB, SAHA or TSA highly increased the number of cells with a reduced ΔΨm (60%, 59% and 79 %, respectively) (Fig. 5a). No reduction of the ΔΨm was observed with cells treated with NaB or SAHA alone, while a weak loss of ΔΨm was found in cells treated with TSA (Fig.5a). Combined treatments also increased the release of cytochrome c and AIF from the mitochondria into the cytosol (data not shown). These results indicate that co-treatments with HDACIs increased the activation of the mitochondrial signalling pathway induced by TRAIL.

### TRAIL/HDACIs co-treatments induce caspases-dependent inactivation of anti-apoptotic proteins

The stability of several pro- and anti-apoptotic proteins was then analysed in SH-EP cells induced by TRAIL and NaB, SAHA or TSA. A strong decrease in the amount of Bim_EL _was observed after 16 h of combined treatments, whereas TRAIL alone induced only a weak reduction of Bim_EL _expression level (Fig. 5b), while Bim_L _and Bim_S _were not detected in SH-EP cells (data not shown). In addition, co-administration of TRAIL and HDACIs increased the down-regulation of the anti-apoptotic proteins XIAP, Bcl-x_L_, survivin, and RIP, compared to TRAIL alone (Fig. 5b and 5c). Interestingly, the kinetic of inactivation of anti-apoptotic proteins was accelerated by the co-treatment with TRAIL and HDACIs. The cleavage of XIAP and the down-regulation of Bcl-x_L _and survivin observed after 8 h of treatment with TRAIL and SAHA was stronger than after 16 h with TRAIL alone (Fig. 5c). Similar results were obtained with TRAIL and NaB (data not shown). Moreover, the kinetic of inactivation of anti-apoptotic proteins is similar to that of caspases activation by proteolytic cleavages (Fig. 4a).

We have previously shown that the down-regulation of XIAP, Bcl-x_L _and RIP induced by TRAIL and chemotherapeutic drugs was caspases-dependent [19]. To analyse if the reduction of the anti-apoptotic proteins XIAP, Bcl-x_L_, survivin, and RIP induced by co-treatments with TRAIL and HDACIs is likewise caspases-dependent, SH-EP cells were treated with TRAIL and NaB, SAHA or TSA in the presence of the pan-caspases inhibitor zVAD. As shown in figure 5d, the cleavage of XIAP, and the inactivation of Bcl-x_L_, survivin, and RIP were protected by the addition of zVAD (Fig. 5d). Similarly, the activation of Bid and caspase-3 induced by TRAIL and HDACIs were protected by zVAD (Fig. 5d). These results indicate that sensitisation to TRAIL by low doses of HDACIs increases the caspases-dependent cleavages and inactivation of anti-apoptotic proteins. In addition, the amount of Bim_EL _was increased in the presence of zVAD indicating that the down-regulation observed by combined treatment was also mediated by caspases-dependent cleavage (Fig. 5d).

### Down-regulation of survivin sensitise NB cells to HDACIs

**Figure 6:**
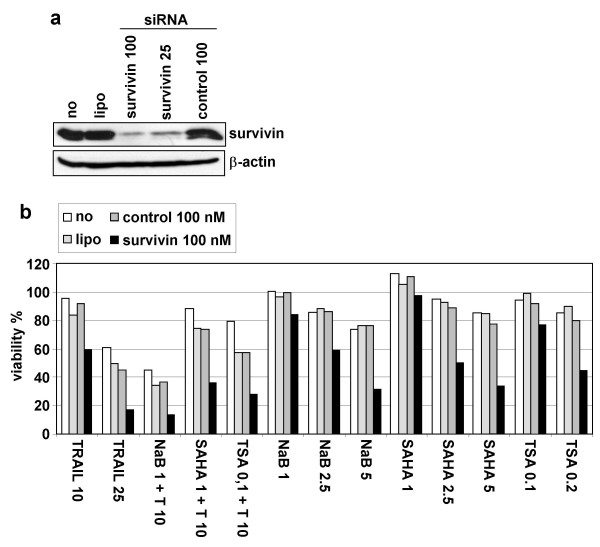
**SiRNAs-mediated down-regulation of survivin expression**. **A** Whole cell extracts of SH-EP cells transfected for 40 h with lipofectamine2000 alone (lipo), with survivin siRNA (25 or 100 nM), control siRNA (100nM), or untransfected (no) were analysed by immunoblotting for the presence of survivin and β-actin as loading control.**B **Survivin down-regulation by siRNAs sensitise NB cells to HDACIs. SH-EP cells were untransfected (no), transfected with lipofectamine 2000 (lipo) alone or with survivin siRNA (100 nM) or control siRNA (100 nM), and treated 40 h after transfection with the indicated amounts of NaB (mM), SAHA, TSA (μM) and TRAIL (ng/ml). Cell death was measured by MTS/PMS cell proliferation assays.

Survivin is over-expressed in most human cancers including NB and was shown to be involved in inhibition of apoptosis in tumour cells [38]. In addition, down-regulation of survivin by siRNAs was shown to sensitise NB cells to TRAIL-induced apoptosis [39]. As survivin steady state level is reduced by co-treatment with TRAIL and HDACIs, we explored its participation in HDACIs-mediated sensitisation to TRAIL. In this aim, survivin expression, was down-regulated in SH-EP cells by RNA interference using survivin siRNAs [36]. As shown in figure 6a, survivin expression level was significantly reduced by survivin siRNA as compared to cells transfected with lipofectamine2000 or control siRNA. The effect of survivin silencing was analysed by proliferation assay (Fig. 6b). Results show that reduction of survivin expression with 100 nM siRNAs sensitised NB cells to TRAIL alone, to low doses of HDACIs as well as to combined treatments with weak doses of TRAIL and HDACIs (Fig. 6b). Similar results were observed with 25 nM of survivin siRNAs (data not shown). These results indicate that down-regulation of survivin induced by HDACIs and TRAIL may account at least in part to the sensitising effect of HDACIs to TRAIL-induced cell death.

## Discussion

The present study demonstrates that simultaneous administration of TRAIL and subtoxic doses of HDACIs strongly potentiates the triggering of apoptotic cascade in NB cells. The detailed analysis of the mechanisms of sensitisation reveals that the increase in cell death is mediated by the enhanced activation of the caspases cascade and the pro-apoptotic protein Bid, concomitant with the down-regulation of the anti-apoptotic proteins RIP, Bcl-x_L_, XIAP, and survivin in a caspases-dependent manner.

Stimulation of the death receptor pathway through enhancement of death receptor expression is one mechanism of sensitisation to TRAIL by HDACIs. Several recent reports have shown that the expression of TRAIL or TRAIL-receptors was induced by HDAC inhibitors in leukaemia cells, breast and colon cancer cells [24-28], while other studies have reported no change in the expression level of DR4, DR5 and DcR2 in melanoma and lymphoma cells [31,37]. We show here that unlike chemotherapeutic drugs, Doxorubicin, Cisplatin, Etoposide or Taxol which up-regulate the cell surface expression of TRAIL-R2 in NB cells [19], HDACIs did not influence any of TRAIL-R1, TRAIL-R2 and TRAIL cell surface expression in NB cells, indicating that sensitisation to TRAIL-induced apoptosis by HDACIs occurs at least partly by different mechanisms.

Several studies reported a change in the expression level of proteins involved in the extrinsic apoptotic pathway such as FADD [24] and FLIP [24,26,29]. No change in the steady state level of caspases-8, -2, -3, -7 and -9, Bid, FADD or FLIP was detected after stimulation with subtoxic doses of NaB, SAHA or TSA in NB cells. Nevertheless, as previously reported in leukaemia cells [31], the death receptor pathway is involved in the synergistic induction of apoptosis by simultaneous treatment with TRAIL and HDACIs in NB cells. Indeed, following over-expression of a FADD-DN protein or c-FLIP_L_, NB cells became fully resistant to TRAIL, which could not be rescued by co-treatments with HDACIs.

In accordance with previous reports [25,26,32,37,40] we demonstrate that HDACIs sensitised NB cells to TRAIL by enhancing the cleavage-mediated activation of the caspases cascade and Bid, while no cleavage was observed with subtoxic doses of HDACIs alone. The enhanced apoptosis induction was caspases-dependent as caspases inhibitors completely abolished NB cell death. Interestingly, TRAIL and HDACIs co-treatments also increased the amplitude of caspases activation. Indeed, a higher level of caspases activities was reached with combined treatment compared to TRAIL alone. This suggests that HDACIs sensitise NB cells to TRAIL-induced apoptosis by enhancing the extent of caspases activation and thereby increasing the magnitude of the apoptotic process.

In addition, the simultaneous addition of TRAIL and HDACIs synergistically affected the mitochondrial pathway as evidenced by the enhanced drop of ΔΨm, the increased caspases-9 activation and cytochrome c and AIF release into the cytosol. This suggests that HDACIs may also act at the level of the mitochondria to sensitise NB cells to TRAIL, as previously reported with other tumour cell lines [24,31,40].

The modulation of the intracellular ratio between pro- and anti-apoptotic proteins could be an other mechanism of HDACIs potentiation to TRAIL-induced apoptosis, which could occur by the increased activity of pro-apoptotic proteins and/or by the down-regulation of anti-apoptotic proteins. The BH3-only protein Bim_EL _was reported to be increased by subtoxic doses of TSA and depsipeptide in CCL and Jurkat cells [32]. Here we show that in NB cells Bim_EL _protein level was reduced by treatment with TRAIL and further decreased by co-administration of HDACIs. This was due to caspases-dependent cleavage as the down-regulation of Bim_EL _was protected by zVAD. Interestingly, the activation of Bim_EL _by caspases-3-dependent cleavage was described to induce a positive feedback amplification of the apoptotic signal by enhancing the affinity of Bim_EL _to Bcl-2 [41]. Therefore, in NB cells Bim_EL _may be activated by caspases-mediated cleavage following combined treatment and such Bim_EL _activation may participate to TRAIL potentiation by HDACIs through the amplification of the apoptotic signal. In addition, we observed the reduction of the anti-apoptotic protein Bcl-x_L _following combined treatments. Hence, the increase of the Bim_EL _/Bcl-x_L _ratio could enhance mitochondrial permeability leading to the release of pro-apoptotic factors.

The caspases-dependent down-regulation of anti-apoptotic proteins such as XIAP and Bcl-2 [31] and the role of the down-regulation of Bcl-x_L _[30] by co-treatment with TRAIL and HDACIs were previously described. In an other report it was shown that the reduction of c-FLIP, Bcl-x_L_, Bcl-2, and XIAP expression induced by subtoxic doses of the HDACI LAQ824 was independent on proteasome or caspases activity [26]. In contrast, we demonstrate here that the steady state level of RIP, Bcl-x_L_, XIAP and survivin in NB cells was reduced by caspases-dependent cleavages mediated by co-treatments with TRAIL and HDACIs. Interestingly, both the level and the timing of down-regulation of anti-apoptotic proteins were increased by combined treatments compared to TRAIL alone. The concomitant caspases-dependent down-regulation of the anti-apoptotic proteins RIP, Bcl-x_L_, XIAP, and survivin, and the activation of the pro-apoptotic proteins Bid and probably Bim_EL _increased the ratio between pro- and anti-apoptotic proteins and therefore lowered the threshold of apoptotic signal and contributed to the sensitising effect of HDACIs to TRAIL-induced apoptosis.

We have previously shown that RIP, Bcl-x_L_, and XIAP were down-regulated by caspases-dependent cleavages upon co-treatment with TRAIL and chemotherapeutic drugs, while the steady state level of survivin was not affected, in contrast to co-treatment with TRAIL and HDACIs [19]. This indicates that HDACIs and chemotherapeutic drugs contribute through different ways to TRAIL-induced apoptosis. The reduction of survivin expression mediated by siRNAs results in an increased the sensitivity threshold of NB cells to HDACIs and/or TRAIL. This suggests that the down-regulation of survivin induced by higher doses of HDACIs and TRAIL plays a role in the sensitising effect of HDACIs to TRAIL.

## Conclusion

The potentiation by HDACIs to TRAIL-induced apoptosis may be caused by stimulation of the apoptotic cascade through increased activation of both the extrinsic and the intrinsic apoptotic pathways induced by TRAIL and HDACIs, respectively. The apoptotic signal is further enhanced by the caspases-dependent activation of pro-apoptotic proteins such as Bid and Bim_el _and inactivation of anti-apoptotic proteins such as XIAP, Bcl-x_L_, RIP, and survivin. It results a change in the equilibrium of pro- to anti-apoptotic molecules that lowers the cell death threshold and strongly favours apoptosis.

These findings may have important implications for the use of TRAIL in cancer therapeutic using recombinant soluble TRAIL. As HDACIs strongly enhance the apoptotic action of TRAIL even at low concentrations, HDACIs may be used in combination with TRAIL to reduce the doses of TRAIL required for inhibition of tumour growth. Recently, several HDAC inhibitors have entered Phase I and Phase II clinical trials and are demonstrating encouraging anti-tumour activity in a variety of cancer types (21). The combination of TRAIL and HDACIs may therefore be an interesting and soft inoffensive new anti-tumour strategy particularly relevant in the treatment of children with highly malignant neuroblastoma.

## Abbreviations

TRAIL: Tumour Necrosis Factor-related apoptosis-inducing ligand, NB: Neuroblastoma, HDACIs: histone deacetylase inhibitors, NaB: sodium butyrate, SAHA: suberoylanilide hydroxamic acid, TSA: Trichostatin A, CHX: cycloheximide, DOX: doxorubicin, DISC: Death Inducing Signalling Complex, c-FLIP: cellular Flice inhibitory protein, IAP: inhibitor of apoptosis protein.

## Competing interests

The author(s) declare that they have no competing interests.

## Authors' contributions

AMM performed all major experimental work including FACS analyses, participated in the design and in the coordination of the study and drafted the manuscript. KBB participated in all cell culture experiments and performed the immunoblots, caspases activity assays. KA participated in the immunoblotting experiment and in cells stimulation with drugs. MF helped for the siRNA transfections. RM participated in cell treatments with HDACIs. JMJ and NG were involved in the overall design of the study and helped to draft the manuscript.

## Pre-publication history

The pre-publication history for this paper can be accessed here:
